# Inositol Hexakisphosphate Inhibits Osteoclastogenesis on RAW 264.7 Cells and Human Primary Osteoclasts

**DOI:** 10.1371/journal.pone.0043187

**Published:** 2012-08-14

**Authors:** María del Mar Arriero, Joana M. Ramis, Joan Perelló, Marta Monjo

**Affiliations:** 1 Department of Fundamental Biology and Health Sciences, Research Institute on Health Sciences (IUNICS), University of Balearic Islands, Palma de Mallorca, Spain; 2 Laboratoris Sanifit, ParcBIT, Palma de Mallorca, Spain; Faculté de médecine de Nantes, France

## Abstract

**Background:**

Inoxitol hexakisphosphate (IP6) has been found to have an important role in biomineralization and a direct effect inhibiting mineralization of osteoblasts in vitro without impairing extracellular matrix production and expression of alkaline phosphatase. IP6 has been proposed to exhibit similar effects to those of bisphosphonates on bone resorption, however, its direct effect on osteoclasts (OCL) is presently unknown.

**Methodology/Principal Findings:**

The aim of the present study was to investigate the effect of IP6 on the RAW 264.7 monocyte/macrophage mouse cell line and on human primary osteoclasts. On one hand, we show that IP6 decreases the osteoclastogenesis in RAW 264.7 cells induced by RANKL, without affecting cell proliferation or cell viability. The number of TRAP positive cells and mRNA levels of osteoclast markers such as TRAP, calcitonin receptor, cathepsin K and MMP-9 was decreased by IP6 on RANKL-treated cells. On the contrary, when giving IP6 to mature osteoclasts after RANKL treatment, a significant increase of bone resorption activity and TRAP mRNA levels was found. On the other hand, we show that 1 µM of IP6 inhibits osteoclastogenesis of human peripheral blood mononuclear cells (PBMNC) and their resorption activity both, when given to undifferentiated and to mature osteoclasts.

**Conclusions/Significance:**

Our results demonstrate that IP6 inhibits osteoclastogenesis on human PBMNC and on the RAW264.7 cell line. Thus, IP6 may represent a novel type of selective inhibitor of osteoclasts and prove useful for the treatment of osteoporosis.

## Introduction

Inositol hexakisphosphate (IP6, phytic acid) is found in high amounts in plant seeds, being their major phosphate store [1], [2]. Afterwards, it has also been shown to be widely distributed in animal cells and tissues [3]–[6]. A large body of evidence has implicated IP6 in a variety of cellular functions such as cell proliferation [7], cell differentiation [8], signal transduction [9], cation transport [10], [11], exocytosis [9], neurotransmission [12], antioxidant [12], efficient transport of mRNA [13] and DNA repair [14].

As regards to biomineralization, different *in vitro* and *in vivo* studies have demonstrated that IP6 is a potent inhibitor of crystallization of calcium salts (oxalate and phosphate salts) [15]–[18]. It has been demonstrated that IP6 inhibits pericardial [19], vascular [20], tooth enamel [21] and renal calcification [22], [23], in addition to inhibiting dental tartar formation [24]. Some results suggest that the mechanism of IP6 in the inhibition of soft tissue calcification is by a reduced hydroxyapatite crystal formation in the first steps, i.e. IP6 would adsorb onto growing crystal faces or avoid nascent crystal nuclei formation, thus impeding further apposition of mineral ions to the crystal [25]–[27]. At the same time, the adsorption of IP6 on critical points of the crystal surface, when already formed, would contribute to its stabilization, thus preventing its dissolution [28]. Therefore, IP6 acts both, preventing the process of formation of calcium salts, but also stabilizing already formed calcium salts, avoiding its subsequent growth and dissolution.

The effect of IP6 on the inhibition of the dissolution of already formed calcium salts is of importance in the prevention of osteoporosis. In agreement with this effect, higher IP6 consumption has been shown to correlate with an increase on bone mineral density (BMD) [29], [30] and with a reduced BMD loss due to estrogen deficiency in an osteoporosis animal model [28]. In fact, IP6 has been proposed to exhibit similar effects to those of non-nitrogen containing bisphosphonates (BP) on bone resorption and to be of use in the primary prevention of osteoporosis [28]. The simplest ones, non–nitrogen-containing BP (such as clodronate and etidronate), can be metabolically incorporated into nonhydrolyzable analogs of ATP that may inhibit ATP-dependent intracellular enzymes resulting in induction of osteoclast apoptosis. The most potent ones, nitrogen-containing bisphosphonates (such as pamidronate, alendronate, risedronate, ibandronate, and zoledronate), can inhibit a key enzyme, farnesyl pyrophosphate synthase, in the mevalonate pathway, thereby preventing the biosynthesis of isoprenoid compounds that are essential for the posttranslational modification of small GTP-binding proteins (GTPases), resulting in the loss of osteoclast activity. Since osteoporosis results from an imbalance between osteoblast and osteoclast (OCL) activity, it is of interest to study the direct effect of IP6 on both types of cells. A recent study by Addison *et al* [31] showed that IP6 inhibits mineralization of MC3T3-E1 osteoblast cultures by binding to growing crystals, increases gene expression of the mineralization inhibitor osteopontin, but does not impair the ability of osteoblasts to synthesize a collagenous matrix, express alkaline phosphatase or differentiate to produce specific bone matrix proteins. However, further investigation is needed to fully understand these effects on osteoblasts and the net effect of IP6 on bone formation, since both in vivo animal and clinical data indicate a positive correlation between IP6 physiological levels and BMD.

The aim of the present study was to investigate the effect of IP6 on the RAW 264.7 monocyte/macrophage mouse cell line and human peripheral blood mononuclear cells (PBMNC) differentiated to OCLs. Undifferentiated and mature OCL treated with IP6 were tested for specific differentiation and functional markers using real-time RT-PCR and TRAP-staining. Activity of OCL was also determined by resorption of dentin discs. To our best knowledge, this is the first study to report the direct effect of IP6 on OCL. Our results demonstrate that IP6 inhibits osteoclastogenesis on human PBMNC and on the RAW 264.7 cell line. Moreover, IP6 showed a differential effect on RAW 264.7 cells depending on when is given in the life stage of OCL, i.e. IP6 inhibits RANKL-induced formation and differentiation from both OCL precursors, mouse RAW 264.7 cell line and human PBMN cells, while further increases differentiation and activity of already mature OCL derived from RAW 264.7 cells, but not those from human primary cells. The biological bases underlying these effects need further investigation.

## Materials and Methods

### Ethics Statement

Human peripheral blood mononuclear cells (PBMNC) were isolated from whole blood of three healthy donors. The samples were obtained after informed consent and with the approval of the Ethical Committee of Balearic Islands (CEI-IB). All participants provided written informed consent.

### Cell Culture

The transformed murine monocytic cell line RAW 264.7 was obtained from ATCC (Manassas, VA, USA). Cells were cultured at 37°C in 5% CO_2_ atmosphere in Dulbecco modified Eagles medium (DMEM) supplemented with 10% fetal bovine serum and antibiotics (50 IU penicillin/ml and 50 µg streptomycin/ml).

Human PBMNC were purified over the Ficoll-Paque following the method described by [32]: 15 mL of whole blood were mixed with 15 ml of warm (37°C) phosphate-buffered saline (PBS, without Ca and Mg), layered over 15 ml of Ficoll-Paque (Amersham Pharmacia Biotech, Uppsala, Sweden) and centrifuged at 400×g for 30 min at room temperature without brake. The cell layer on top of the Ficoll-Paque was carefully collected and washed two times with α-minimum essential medium (α-MEM;Gibco, Grand Island, NY, US) containing 10% fetal bovine serum (HyClone). Subsequently, cells were counted and used immediately. The procedure was performed in triplicate for each donor.

### Cytotoxicity Assay

RAW cells 264.7 were seeded at a density of 20,000 cells/cm^2^ in 24-well plate and cultured for 24 h. After cell attachment, culture media was changed and different doses of IP6 were added. Cells were cultured for additional 24 hours and culture media was collected to test cytotoxicity (LDH activity). LDH cytotoxicity assay was performed according to the manufacturer’s protocol (Cytotoxicity Detection Kit (LDH), Roche Diagnostics, Switzerland). This colorimetric assay quantifies activity of LDH released from the cytosol of damaged cells into the supernatant and thus serves as an index of cell death.

Results were presented relative to the LDH activity in the media of control cells (100% of cell viability) and of cells treated with 1% Triton X-100 (0% cell viability) using the equation: Cell viability (%) = (IP6-treated cells−Control cells)/(Triton-treated cells−control cells)×100.

### Cell Proliferation Assay

RAW cells 264.7 were seeded at a density of 2,500 cells/well in a 96-well plate and cultured for 24 h. After cell attachment, culture media was changed and different doses of IP6 were added. Cells were cultured for additional 24 hours and bromodeoxyuridine (BrdU) was added for the last 6 hours. Incorporation of BrdU was determinated by Cell Proliferation ELISA kit as described by the manufacturer (Roche Diagnostics, Switzerland).

### Generation of Osteoclasts

Osteoclast-like cells (OCL) were generated in culture from RAW 264.7 cells or from human PBMNC.

RAW 264.7 cells were seeded in 24-well plates at a density of 20,000 cells/cm^2^ and placed in the CO_2_ incubator overnight to allow the cells to attach to the surface. After 24 h, the culture medium was replaced with media containing 100 ng/mL RANKL (PeproTech, Rocky Hill, NJ, USA). Osteoclasts were successfully generated by dosing with RANKL every 48 h over the course of 5 days.

PBMNC were seeded in 96-well plates at a density of 1.85×10^6^ cells/cm^2^. After 2 hours of incubation, cells were washed to remove non-adherent cells. Then, cells were cultured with media (alpha-MEM containing 10% FBS and 1% penicillin/streptomycin) supplemented with 25 ng/ml M-CSF (R&D Systems, Minneapolis, MN, USA), 50 ng/ml RANKL (R&D Systems, Minneapolis, MN, USA) and 1 µM dexamethasone (Sigma-Aldrich, St.Louis, MO, USA) as previously described [32]. The cells were re-fed twice weekly by demi-depletion (half of the medium was replaced with fresh medium) and cultured for up to 21 days.

To confirm the generation of multinucleated osteoclast-like cells, the cultured cells were stained for the enzyme tartrate-resistant acid phosphatase (TRAP) using the TRAP-staining kit (Sigma-Aldrich, St.Louis, MO, USA), according to the manufacturer’s instructions. TRAP is the enzyme that has been used as a marker of osteoclast function for more than 20 years [33]. TRAP-positive multinucleated (3 or more nuclei) osteoclasts were visualized by light microscopy and photographed. Each OCL formation assay was performed at least 3 times.

### Effect of IP6 on OCL Formation

To examine the effect of IP6 on OCL formation, RAW 264.7 cells were seeded at 20,000 cells/cm^2^ density and, after an overnight period, the culture medium was replaced with media containing 100 ng/mL RANKL and different doses of IP6 (0.1, 1, 10, 100 µM). Treatments were added after changing the media every 48 h over the course of 5 days.

To examine the effect of 1 µM IP6 on OCL formation from human PBMNC, purified cells were seeded at a density of 1.85×10^6^ cells/cm^2^ and, after 2 h of incubation, cells were treated with media containing 1 µM of IP6 and 25 ng/ml M-CSF, 50 ng/ml RANKL and 1 µM dexamethasone. Treatments were added on each changing media over the course of 21 days.

The effect of IP6 on OCL formation was assessed by analysis of the number of TRAP-positive cells with 3 or more nuclei, gene expression levels of osteoclast and functional markers, resorption activity on dentin discs.

### Effect of IP6 on Mature Osteoclasts-like Cells

OCL were generated by dosing RAW 264.7 cells with RANKL over the course of 5 days as described above. OCL generated from human PBMNC were generated by dosing the cells with 25 ng/ml M-CSF, 50 ng/ml RANKL and 1 µM dexamethasone for 21 days. Then, on the fifth day for RAW cells and on the 21^st^ day for PBMNC, OCLs were treated with IP6 (1 µM) for 24 hours (RAW cells) or for 4 days (PBMNC). The effect of IP6 on mature OCLs generated from RAW cells was assessed by analysis of the number of TRAP-positive cells, gene expression levels of osteoclast and functional markers and resorption activity on dentin discs. The effect of IP6 on mature human OCLs was assessed by resorption activity on dentin discs.

### RNA Isolation and RT-PCR Analysis

Total RNA was isolated with Tripure (Roche Diagnostics) from cells treated as describe above, following the instructions of the manufacturer. RNA was quantified using a spectrophotometer set at 260 nm (Nanodrop, Thermo Fisher Scientific Inc, US).

The same amount of total RNA (1 µg) from each sample was reverse transcribed to cDNA at 37°C for 60 minutes in a final volume of 20 µl, using High Capacity RNA to cDNA kit (Applied Biosystems, USA). Each cDNA was diluted 1/5 and aliquots, to avoid freezing and thawing cycles, were stored at −20°C until the PCR reactions were carried out.

Real-time PCR was performed for two reference genes: 18S ribosomal RNA (18S rRNA), glyceraldehyde-3-phosphate dehydrogenase (*Gapdh*); three osteoclast gene markers: tartrate resistant acid phosphatase (*Trap*), calcitonin receptor (*CalcR*), colony stimulating factor receptor *(Cfms*); and four osteoclast functional markers: cathepsin K (*CtsK*), metalloproteinase-9 (*Mmp-9*), carbonic anhydrase type II *(Car2*) and vacuolar-type H^+^ ATPase (subunit Atp6v0d2) (*H^+^-ATPase*). Primer sequences are shown in Table 1.

**Table 1:** Oligonucleotide primer pairs used for real-time RT-PCR.

Gene	GenBank ID	Sequences
***mGapdh***	XM_132897	Forward:5′-CCCAGAAGACTGTGGATGG-3′Reverse: 5′-CAGATTGGGGGTAGGAACAC-3′
***m18S rRNA***	X00686	Forward: 5′-GTAACCCGTTGAACCCCATT-3′Reverse: 5′-CCATCCAATCGGTAGTAGCG-3′
***mTrap***	NM_007388.2	Forward: 5′-GCGACCATTGTTAGCCACATACG-3′Reverse: 5′-CGTTGATGTCGCACAGAGGGAT-3′
***mCalcR***	NM_001042725	Forward: 5′-TGGTGCGGCGGGATCCTATAAGT-3′Reverse: 5′-AGCGTAGGCGTTGCTCGTCG-3′
***mCfms***	NM_001037859	Forward: 5′-TGGATGCCTGTGAATGGCTCTG-3′Reverse: 5′-GTGGGTGTCATTCCAAACCTGC-3′
***mCtsk***	NM_007802.3	Forward: 5′-AGCAGAACGGAGGCATTGACTC-3′Reverse: 5′-TTTAGCTGCCTTTGCCGTGGC-3′
***mMmp-9***	NM_013599.2	Forward: 5′-GCTGACTACGATAAGGACGGCA-3′Reverse: 5′-GCGGCCCTCAAAGATGAACGG-3′
***mCar2***	NM_009801.4	Forward: 5′-CTCTGCTGGAATGTGTGACCTG-3′Reverse: 5′-CTGAGCTGGACGCCAGTTGTC-3′
***mH^+^ATPase (Atp6v0d2)***	NM_175406.3	Forward: 5′-ACGGTGATGTCACAGCAGACGT-3′Reverse: 5′-CCTCTGGATAGAGCCTGCCGCA-3′

Real-time PCR was performed in the Lightcycler 480® (Roche Diagnostics, Mannheim, Germany) using SYBR green detection. Each reaction contained 7 µl Lightcycler-FastStart DNA MasterPLUS SYBR Green I (containing Fast Start Taq polymerase, reaction buffer, dNTPs mix, SYBRGreen I dye and MgCl2), 0.5 µM of each, the sense and the antisense specific primers (Table 1) and 3 µl of the cDNA dilution in a final volume of 10 µl. The amplification program consisted of a preincubation step for denaturation of the template cDNA (5 min 95°C), followed by 45 cycles consisting of a denaturation step (10s 95°C), an annealing step (10s 60°C) and an extension step (10s 72°C). After each cycle, fluorescence was measured at 72°C. A negative control without cDNA template was run in each assay.

To allow relative quantification after PCR, standard curves were constructed from standard reactions for each target and housekeeping genes. The crossing point readings for each of the unknown samples were used to calculate the amount of either the target or housekeeping relative to a standard curve, using the Second Derivative Maximum Method. Relative mRNA levels were calculated as the ratio of relative concentration for the target genes in the same sample using the Advanced Relative Quantification Method provided by the LightCycler 480 analysis software version 1.5 (Roche Diagnostics, Mannheim, Germany).

### Resorption Pit Assay

RAW 264.7 cells were seeded on dentine slices (Immunodiagnostic Systems, Boldon, UK) at a density of 20,000 cells/cm^2^. To study the effect of IP6 on OCL formation cells were cultured with media containing 100 ng/mL RANKL and IP6 (1 µM) over the course of the entire experiment. To study the effect of IP6 on mature osteoclasts-like cells, OCL were generated by dosing RAW 264.7 cells with RANKL over the course of 6 days and then treated with IP6 (1 µM) for 4 days.

Human PBMNC seeded on dentine discs (1.85×10^6^ cells/cm^2^) were treated with 25 ng/ml M-CSF (R&D Systems, Minneapolis, MN, USA), 50 ng/ml RANKL (R&D Systems, Minneapolis, MN, USA) and 1 µM dexamethasone (Sigma-Aldrich, St.Louis, MO, USA). To study the effect of 1 µM IP6 during osteoclastogenesis, cells were treated with IP6 for 21 days, and to study the effect of 1 µM IP6 on mature osteoclasts, cells were treated with IP6 for 4 days after 21 days of culture.

After the culturing period, cells were removed from the dentine slices by sonication in 0.1 N NaOH for 2 minutes, stained in hematoxylin for 40 seconds and washed in distilled water. The surface of each dentine slice was examined by light microscopy for evidence of lacunar resorption and quantitative analysis of the resorption area was performed with Image J software 1.44p (NIH, USA).

### Statistical Analysis of Data

For experiments performed with RAW cells, the data were presented as mean values ± SEM (n = 6). Differences between groups were assessed by Mann-Whitney-test or by Student t-test depending on their normal distribution. For experiments performed with human PBMNC, three different donors were used and the experiments were performed in triplicate, for each donor the data were presented as mean values ± SEM. Differences between groups were assessed by paired t-test. Data analysis was performed using a statistical software package (SPSS, Chicago, IL, US). Results were considered statistically significant at the p-values ≤0.05.

## Results

### RAW 264.7 Cells Exhibit Unchanged Viability and Proliferation in the Presence of IP6

**Figure 1:**
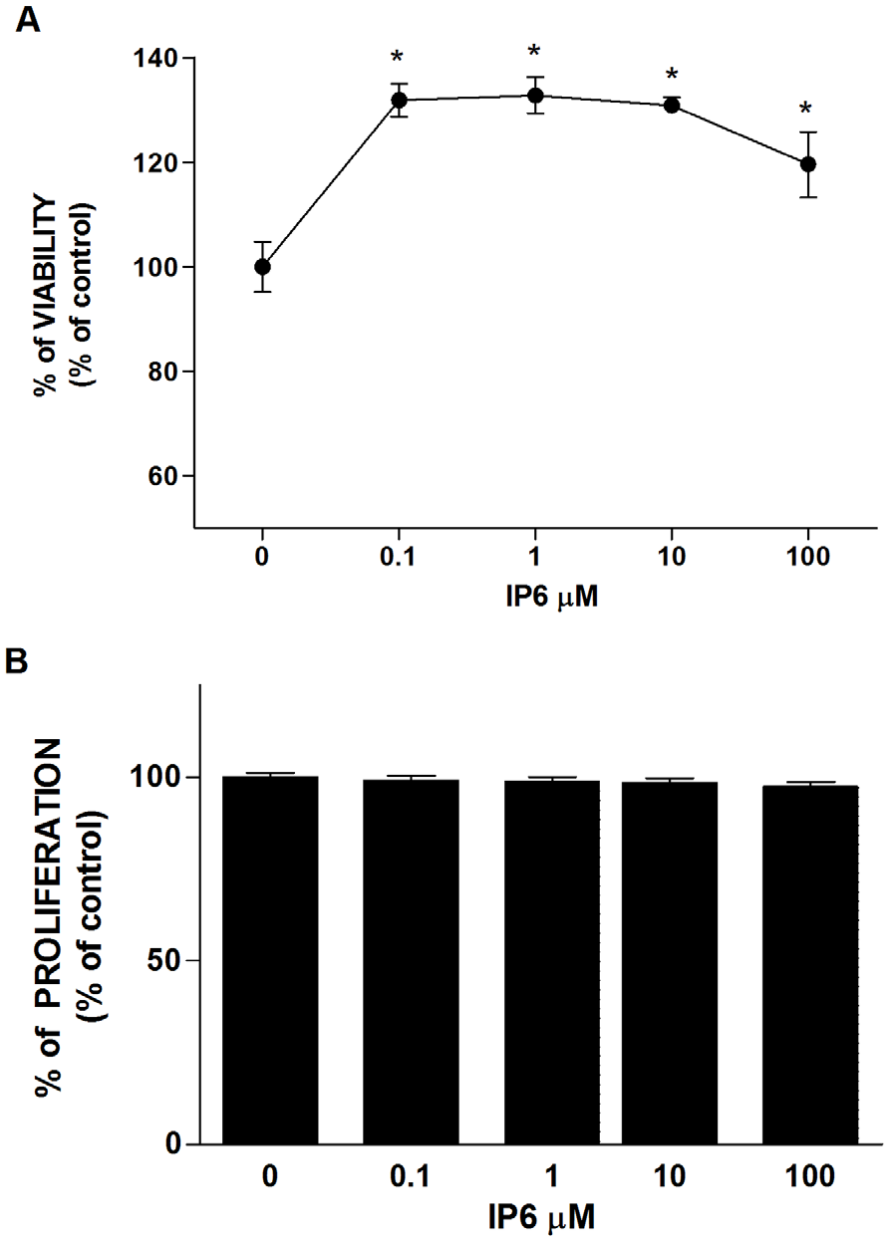
Effect of inositol hexakisphosphate (IP6) on cell viability and proliferation of osteoclast progenitors cells. (A) LDH activity measured from culture media collected after treatment of cells with different doses of IP6 for 24 hours. Results are presented relative to the LDH activity in the media of control cells (100% of cell viability) and of cells treated with 1% Triton X-100 (0% cell viability) using the equation described in Materials and Methods section. Values represent the mean ± SEM. Significant differences were assessed by Mann-Whitney test: *p≤0.05 versus untreated cells (n = 6). (B) Proliferation of RAW 264.7 cells treated with different doses of IP6 for 24 hours and labeled with BrdU for 6 h. Values are expressed as a percentage of control cells, which were set to 100%. Values represent the mean ± SEM. Significant differences were assessed by Student’s t test (n = 6).

First, we determined the effect of increasing concentrations of IP6 on cell viability of RAW 264.7 cells, using LDH activity as an index of cell death. As seen in figure 1A, IP6 demonstrated no cytotoxic effects after 24 hours of treatment at any of the doses tested. Remarkably, IP6 showed a protective effect on osteoclast precursors against cell damage compared to untreated cells.

The resulting effects of the IP6 on cell proliferation are shown in Figure 1B. BrdU incorporation showed that IP6 treatment had no effects on cellular proliferation within the concentration range of 0.1–100 µM in RAW 264.7 cells after 24 h of treatment.

### IP6 Directly Inhibits Osteoclast Formation and Gene Expression of Phenotypic and Functional Markers Induced by RANKL

**Figure 2:**
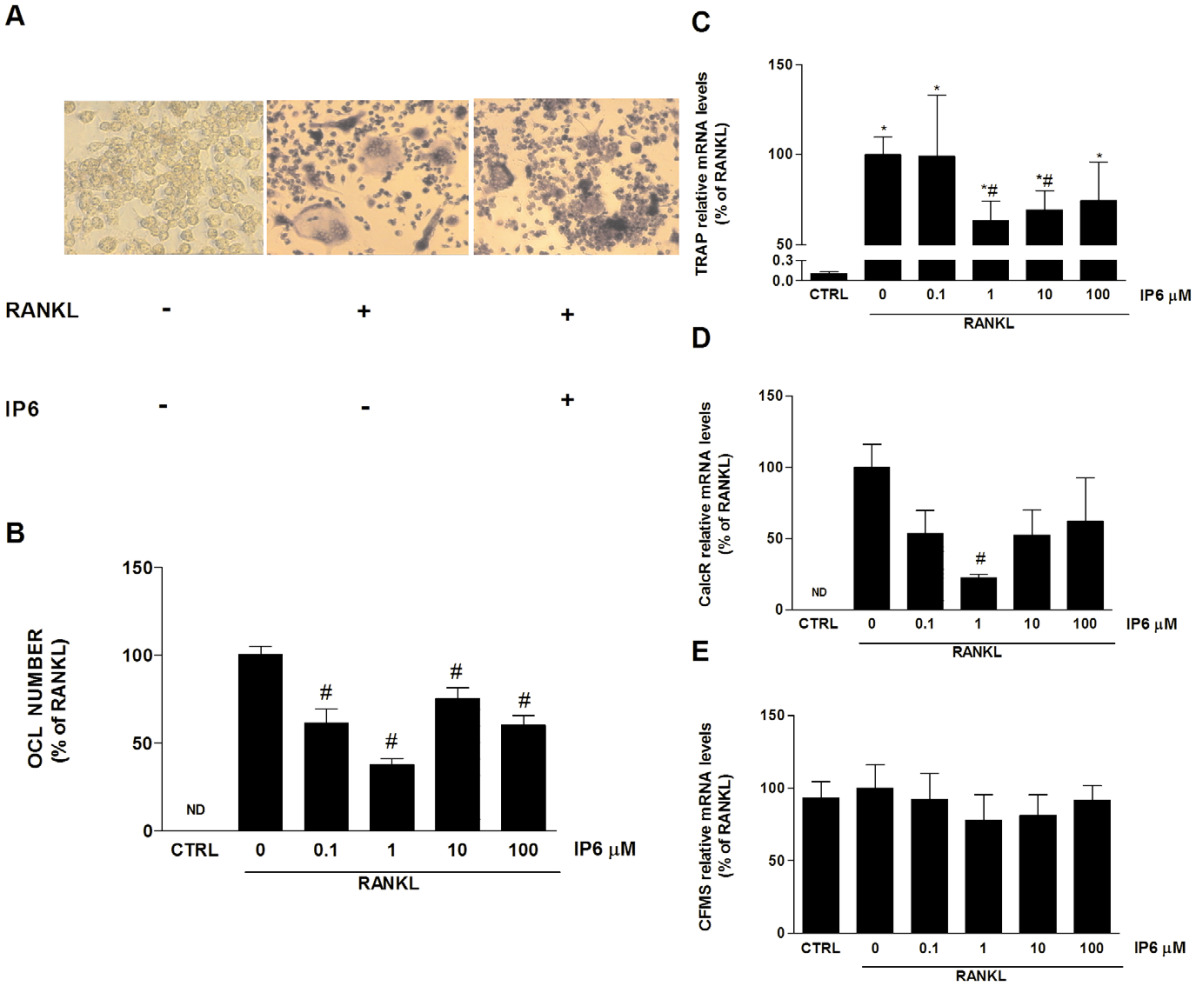
Inositol hexakisphosphate (IP6) directly inhibits osteoclast formation induced by RANKL. (A) Effect of IP6 treatment on the generation of multinucleated TRAP-positive cells (OCL). RAW 264.7 cells cultured for 5 days with no stimulation with RANKL (left). RAW 264.7 dosed with 100 ng/mL RANKL for 5 days (center). RAW 264.7 cells dosed with 100 ng/mL RANKL and treated with 1 µM IP6 for 5 days (right). Representative images are shown. (B) Number of multinucleated TRAP-positive cells with 3 or more nuclei (OCL) generated from RAW264.7 treated with IP6. Values are expressed as a percentage of RANKL-dosed cells non-treated with IP6, which was set to 100.*(n = 3)* (C) *Trap* mRNA levels, (D) *Cfms* mRNA levels and (E) *CalcR* mRNA levels of RANKL-stimulated cells and treated with IP6. Data represent fold changes of target genes normalized with *Gapdh* mRNA and 18s rRNA, expressed as a percentage of RANKL-dosed cells non-treated with IP6, which were set to 100%. Values represent the mean ± SEM. Significant differences were assessed by Student’s t test: #p≤0.05 versus RANKL treated cells. (n = 6).

To investigate the effects of IP6 on osteoclastogenesis, RAW 264.7 cells were treated from the first day of the experiment with different doses of IP6 (0.1–100 µM) in the presence of RANKL (100 ng/ml), as indicated in the methods section. After the fifth day of incubation, cells were processed for TRAP staining and counting of multinucleated cells related to the total number of cells. As seen in figure 2A, staining confirmed that the presence of TRAP-positive multinucleated osteoclast-like cells was limited to those wells dosed with exogenous RANKL, under the cell culture conditions described. Treatment with 1 µM of IP6 decreased the number of OCL formed and their morphology, with smaller OCL and with fewer nuclei than cells non-treated with IP6. The number of multinucleated (with 3 or more nuclei) TRAP-positive cells (OCL) generated from RAW264.7 treated with IP6 is presented in figure 2B. RAW 264.7 cells dosed with RANKL and treated with IP6 at different doses showed significantly lower number of TRAP-positive multinucleated cells than those dosed with RANKL and non-treated with IP6, showing a major effect the 1 µM dose of IP6, reducing OCL number induced by RANKL by approximately 40%. These results may indicate that IP6 has a biphasic effect on osteoclastogenesis, since concentrations higher than 1 µM become less effective.

We also assessed the effect of IP6 on osteoclastogenesis by evaluating gene expression levels of several OCL phenotypic markers (Figure 2 C, D and E). Throughout the differentiation process, OCL express a series of markers, such as tartrate-resistant acid phosphatase (*Trap)*, the receptor for macrophage-colony-stimulating factor (*Cfms*), and the calcitonin receptor (*CalcR*), which, along with multinucleation and resorption, characterize the OCL phenotype. TRAP is expressed in high levels in the osteoclasts, being used as a marker of OCL function [33]. Mature OCL also express the calcitonin receptor upon its surface, in fact, it has been described as the best differentiation marker for the OCL [34]. Last, CFMS triggers the proliferation and fusion of mononuclear cells and the formation of multinucleated, mature osteoclasts [35].

*Trap* mRNA expression levels were significantly higher in all groups dosed with RANKL (Fig. 2C). Moreover, a decrease on *Trap* mRNA levels was found on cells treated with IP6, although only statistical significance was reached by cells treated with 1 µM of IP6. Similar results were found for *CalcR* mRNA expression levels (Fig. 2D). IP6 treatment decreased *CalcR* mRNA levels compared to RANKL dosed-cells, but just 1 µM of IP6 was statistically significant. For the group of cells no dosed with RANKL, *CalcR* mRNA was not detectable. As seen in Fig. 2E, no differences were found on *Cfms* mRNA expression levels between the different groups analyzed, most probably due to the fact that RAW 264.7 cells do not require M-CSF in their RANKL-induced formation into mature OCL and consequently, M-CSF was not used in the experiments.

**Figure 3:**
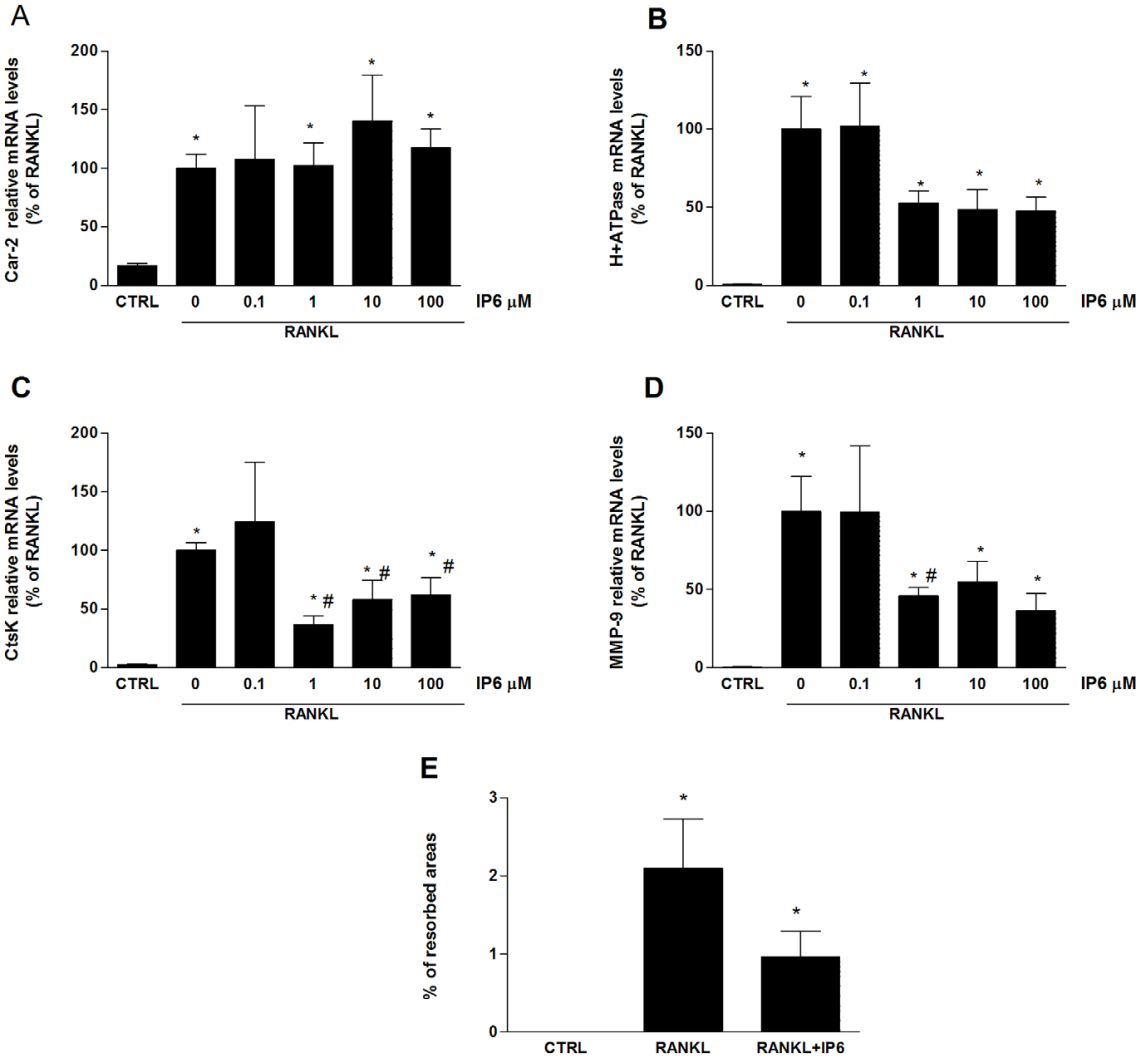
IP6 directly inhibits RANKL-induced osteoclast bone resorption ability. RAW 264.7 cells were treated with RANKL (100 ng/ml) for the generation of OCL and IP6 for 5 days and gene expression of osteoclast functional markers was determined: *Car-2*(A), *H^+^-ATPase* (B), *CtsK* (C) and *Mmp-9* (D). Data represent fold changes of target genes normalized with GAPDH mRNA and 18s rRNA, expressed as a percentage of RANKL-dosed cells non-treated with IP6, which were set to 100%. Values represent the mean ± SEM. Significant differences were assessed by Student’s t test: *p≤0.05 versus control cells. #p≤0.05 versus RANKL treated cells. (n = 6) (E) Bone resorption ability of RAW 264.7 cell treated with 1 µM of IP6 during osteoclastogenesis was evaluated by resorption pit assay on dentine discs (n = 3). Data represent the percentage of the resorbed area by osteoclasts. Values represent the mean ± SEM. Significant differences were assessed by Mann-Whitney test:*p≤0.05 versus untreated cells. #p≤0.05 versus RANKL treated cells.

We next evaluated the bone resorptive activity of OCL generated from RAW 264.7 cells treated with RANKL and IP6 by measuring gene expression levels of markers related to OCL activity. When osteoclasts are attached to bone matrix, carbonic anhydrase type II (*Car2*) generates H^+^ and HCO_3_^−^ from the hydration of CO_2_. The protons are transported through the apical ruffled border to the resorption zone by a vacuolar type proton ATPase *(H^+^ATPase*). After solubilization of the mineral phase, several proteolytic enzymes degrade the organic bone matrix. The high levels of expression of *Mmp-9* (gelatinase B) and cathepsin K (*CtsK*) and of their secretion into the resorption lacuna suggest that these enzymes play a central role in the resorption process [23], [24]. *Car-2* mRNA levels increased in OCL treated with RANKL but no effect was found after IP6 treatment at any of the doses tested (Fig. 3A). However, IP6 at doses ranging from 1 to 100 µM decreased mRNA levels of *H^+^ATPase*, *CtsK* and *Mmp-9* (Fig. 3B–D), with significant values for *CtsK* (1–100 µM) and *Mmp-9* (1 µM).

Then, we tested the ability of OCL generated from RAW 264.7 cells and treated with RANKL (100 ng/ml) and IP6 (1 µM) to resorb dentin discs (Fig. 3E). A decrease in the percentage of resorbed areas in the dentin discs after IP6 treatment was observed, although statistical significance was not reached.

### IP6 Directly Stimulates Gene Expression of Osteoclast Phenotypic and Functional Markers and Resorption Activity on Mature Osteoclasts-like Cells

**Figure 4:**
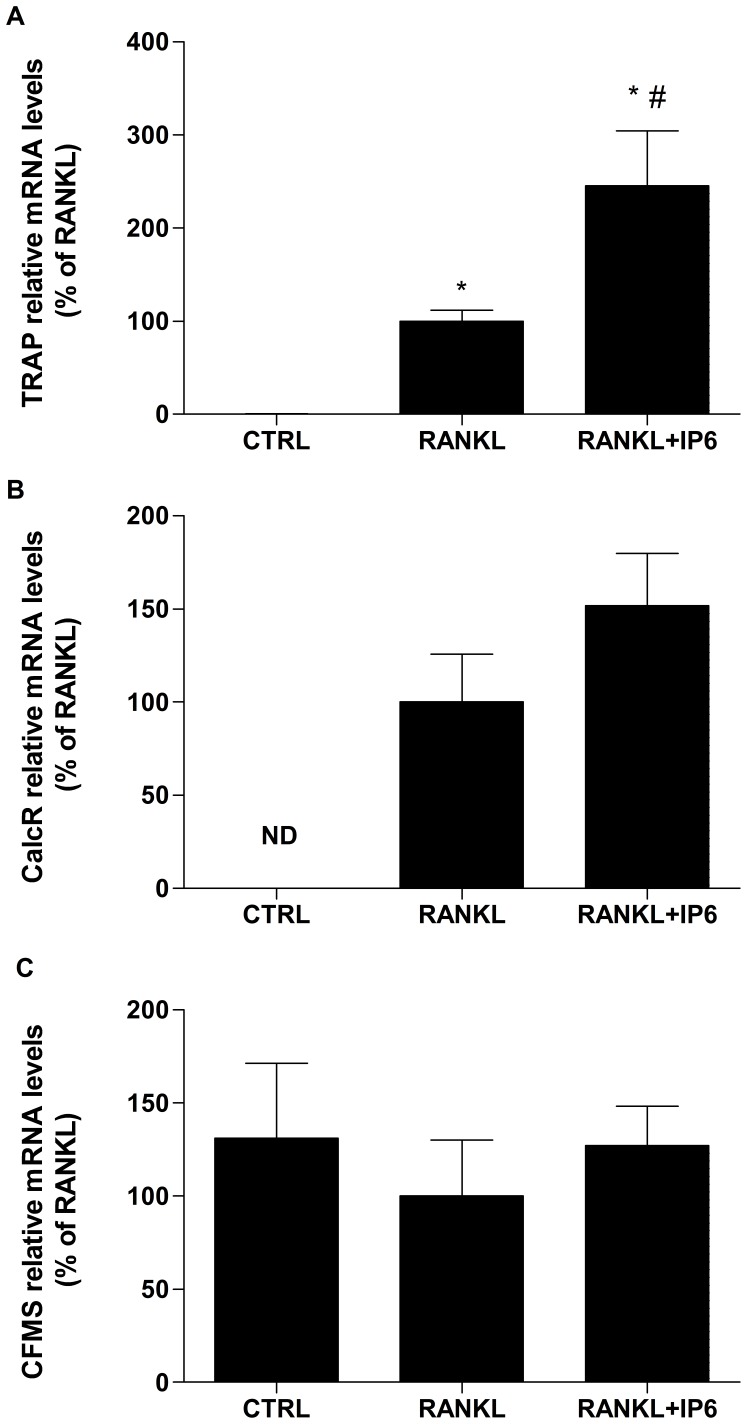
IP6 directly stimulates gene expression of osteoclast phenotypic markers on mature osteoclasts-like cells. Mature osteoclasts were treated with 1 µM of IP6 for 24 hours and gene expression of osteoclast phenotypic markers was determined*: Trap* (A*), CalcR* (B) and *Cfms*(C). Data represent fold changes of target genes normalized with *Gapdh* mRNA and 18s rRNA, expressed as a percentage of RANKL-dosed cells non-treated with IP6, which were set to 100%. Values represent the mean ± SEM. Significant differences were assessed by Student’s t test: *p≤0.05 versus control cells; #p≤0.05 versus RANKL treated cells. (n = 6).

We assessed the effect of treatment for 24 hours with 1 µM IP6 on OCL generated from RAW 264.7 cells by evaluating gene expression levels of osteoclast phenotypic markers. *Trap* mRNA levels were significantly higher in cells treated with IP6 compared to both, OCL non-treated and to precursor cells non-dosed with RANKL (Fig. 4A). Similar results were found for *CalcR* mRNA expression levels (Fig. 4B), although data were not statistically significant. No differences were found on CFMS mRNA levels after IP6 treatment, similar to earlier experiments (Fig. 4C).

**Figure 5:**
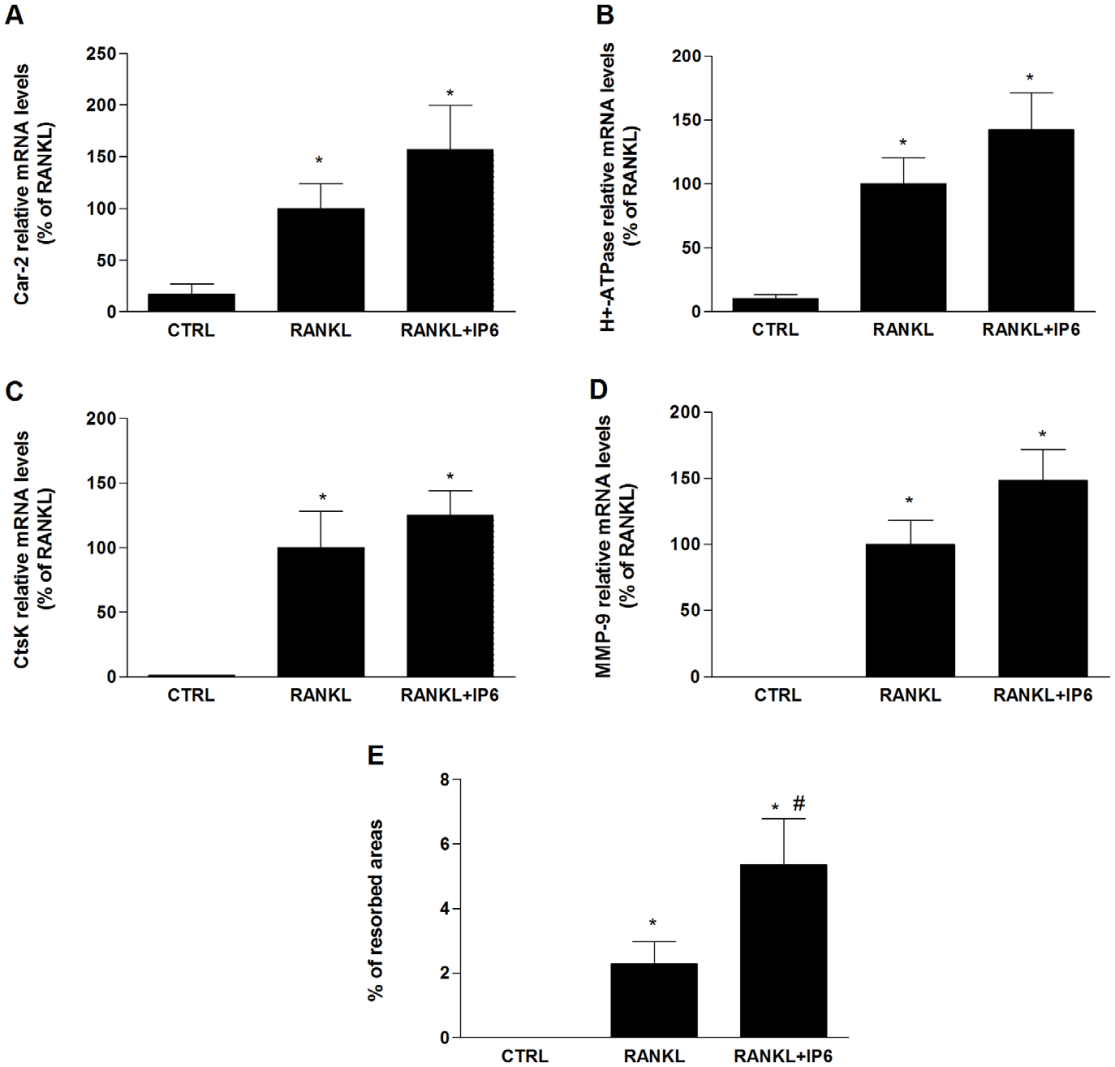
IP6 directly stimulates gene expression of osteoclast functional markers and resorption activity on mature osteoclasts-like cells. Mature osteoclasts were treated with 1 µM of IP6 for 24 hours and gene expression of osteoclast functional markers was determined: *Car-2*(A), *H^+^-ATPase* (B), *CtsK* (C) and *Mmp-9* (D). Data represent fold changes of target genes normalized with *Gapdh* mRNA and 18s rRNA, expressed as a percentage of RANKL-dosed cells non-treated with IP6, which were set to 100%. Values represent the mean ± SEM. Significant differences were assessed by Student’s t test: *p≤0.05 versus control cells. (E) Bone resorption ability of mature osteoclasts treated with 1 µM of IP6 for four days was evaluated by resorption pit assay on dentine discs. Data represent the percentage of the resorbed area by mature osteoclasts. Values represent the mean ± SEM. Significant differences were assessed by Student’s t test: *p≤0.05 versus untreated cells. #p≤0.05 versus RANKL treated cells. (n = 3).

We further examined whether IP6 had an effect on gene expression of osteoclast functional markers (Fig. 5A–D). The four functional osteoclast markers studied showed a significant increase after IP6 treatment for 24 hours on mature OCL compared to RAW 264.7 cells no dosed with RANKL (Fig. 5A–D). Although OCL treated with IP6 showed higher expression levels of the analyzed functional markers compared to untreated mature OCL, differences were not significant.

We next evaluated the ability of mature OCL treated with IP6 to resorb bone. RAW 264.7 cells were plated on dentine slices and stimulated with RANKL (100 ng/ml) for five days and treated for another four days in the presence or absence of 1 µM of IP6. RANKL-stimulated cells formed a number of pits, suggesting that the bone resorption activity of RANKL-treated cells made them into functionally active state-resembling OCL. In agreement with our previous results with mature OCL, treatment with 1 µM of IP6 significantly increased the resorbed area compared with treatment with RANKL alone (Fig. 5E).

### IP6 Inhibits Osteoclastogenesis of Human PBMNC and their Resorption Activity

Since the RAW264.7 cell line remains a model system, to provide a more comprehensive evaluation of the effects of IP6 in a more physiological system, we investigated the effect of IP6 on human primary osteoclasts.

**Figure 6:**
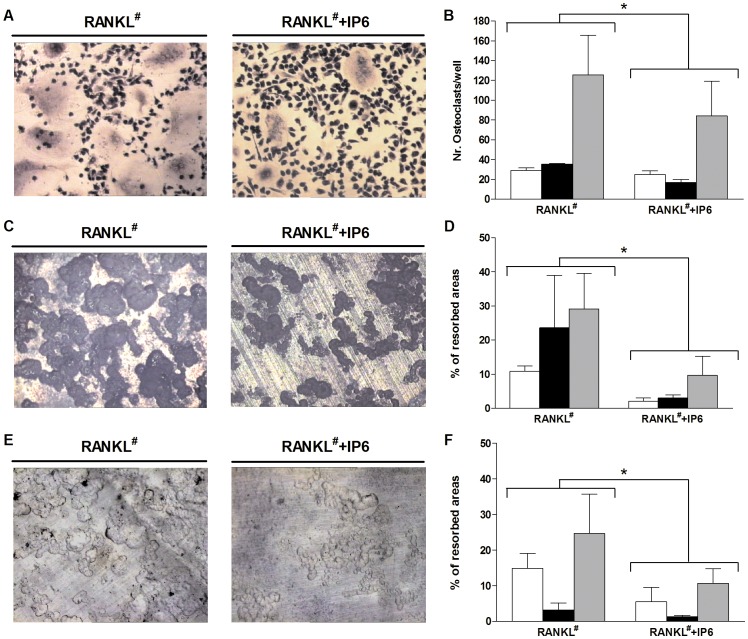
Effect of inositol hexakisphosphate (IP6) on human peripheral blood mononuclear cells (PBMNC) osteoclastogenesis and resorption activity on mature osteoclasts cells derived from human PBMNC. (A) Effect of IP6 treatment on the generation of multinucleated TRAP-positive cells (OCL). Human PBMNC, from three different donors, were cultured for 21 days with RANKL^#^ (left) and with RANKL^#^ plus 1 µM IP6 for 21 days (right). RANKL^#^ refers to cells treated with RANKL and also with M-CSF and dexamethasone as described in the Materials & Methods section. Representative images are shown. (B) Number of multinucleated TRAP-positive cells with 3 or more nuclei (OCL) generated from human PBMNC treated with IP6. (C) Bone resorption ability of human PBMNC treated with RANKL^#^ alone (left) and with 1 µM of IP6 during osteoclastogenesis (right) was evaluated by resorption pit assay on dentine discs (D) Percentage of resorbed area by human PBMNC treated with or without IP6 (1 µM) for each donor. (E) Representative images of bone resorption by mature osteoclasts treated with RANKL^#^ alone (left) and with IP6 (right). (F) Percentage of resorbed dentin area by mature osteoclasts. Three different donors were used and the experiments were performed in triplicate, different shaded bars represent different donors, for each donor the data were presented as mean values ± SEM. Significant differences were assessed by paired t- test: *p≤0.05 versus RANKL^#^ treated cells.

Human PBMNC obtained from tree different donors were treated from the first day of the experiment with 1 µM of IP6 in the presence of M-CSF (25 ng/ml), RANKL (50 ng/ml) and dexamethasone (1 µM) for 21 days. As seen in figure 6, treatment with 1 µM IP6 significantly decreased (Figure 6, A–B) the number of TRAP-positive multinucleated osteoclasts (p = 0.013) and (Figure 6, 6C–D) their ability to resorb dentin discs (p = 0.015), in agreement with the results obtained with the RAW264.7 cell line.

However, when IP6 treatment was given to mature osteoclasts derived from human PBMNC (Figure 6, E–F), in contrast to the results obtained with the RAW264.7 cell line, a significant decrease in the resorbed area on dentine discs (p = 0.032) was observed.

## Discussion

This study shows for the first time that IP6 inhibits osteoclastogenesis on human PBMNC and on the RAW264.7 cell line. For the present investigation, human primary osteoclasts and murine macrophage RAW 264.7 cells were used to study the direct effect of IP6 on osteoclastogenesis and activity of mature OCL. RAW 264.7 cells respond to RANKL stimulation *in vitro* to generate bone pit resorptive multinucleated OCL with the hallmark characteristics expected for fully differentiated OCL [36]–[38]. These cells are in a pre-osteoclast state and only RANKL is needed for osteoclast differentiation [39], [40]. RAW 264.7 cells express the CFMS receptor for M-CSF [41] as well as M-CSF, perhaps explaining why they also express high levels of RANK [36] and do not require M-CSF as a permissive factor in their RANKL-induced formation into mature OCL. In the present study, no differences were found in CFMS receptor expression after RANKL and IP6 treatment, as M-CSF was not added to the biological system during the experimental procedure.

Since the RAW264.7 cell line remains a model system, to provide a more comprehensive evaluation of the effects of IP6 in a more physiological system, we investigated the effect of IP6 on human primary osteoclasts. Mononuclear cells isolated from human peripheral blood provides a cell source capable to differentiate into osteoclasts when cultured with RANKL, M-CSF and dexamethasone [32]. Therefore, primary cultures of human PBMNC stimulated to osteoclasts differentiation were chosen to verify the effect of IP6 found on RAW264.7 cells. However, variables among different donors must be taken into account when using primary cells; to overcome this problem and to improve validity of our data, we have used three different donors.

Bone is continually remodeled through the synthesis of new bone by osteoblasts and its resorption by OCL. In osteoporosis, this equilibrium is disrupted in favor of increased activity of OCL, without the compensatory synthesis of new bone by osteoblasts [42]. Bisphosphonates (BP) are so far the most commonly used antiresorptive drugs in osteoporosis therapy [43], [44]. They are synthetic analogs of pyrophosphate with a strong affinity for Ca^2+^ ions of hydroxyapatite that have been demonstrated to prevent or disrupt calcium salt crystallization and dissolution in the same way as IP6 [28]. Apart from the effect on hydroxyapatite dissolution, BP have been shown to inhibit osteoclast formation and resorption activity [45]–[48], and they also influence important cellular functions of osteoblasts such as cell proliferation and differentiation, expression and secretion of cytokines, synthesis of extracellular matrix proteins, and synthesis and secretion of OPG and RANKL [49]–[53].

IP6 has only been reported to have an inhibitory effect on mineralization in osteoblastic cells, and to the best of our knowledge, no studies have documented the direct effect on OCL. We show that IP6 reduces osteoclast formation without affecting RAW264.7 cell proliferation or viability. The effects found of IP6 were not due to toxic effects on OCL precursors, as shown by the low LDH activity in the IP6-treated cells. Thus, IP6 does not appear to act through a cytotoxic mechanism.

We also investigated whether IP6 could have an effect on the proliferation of OCL precursors. The treatment with IP6 had no effects on cell proliferation within the concentration range of 0.1–100 µM. Thus, a difference of IP6 compared to BP is the lack of inhibition on proliferation of OCL precursors. Although IP6 did not affect cell proliferation, it decreased osteoclastogenesis of human primary osteoclasts and of RAW 264.7 cells.

More importantly, in RAW 264.7 cells IP6 was also found to decrease the RANKL-induced up regulation of markers related to OCL activity, H^+^-ATPase, MMP-9 and Cathepsin K. For digestion and solubilization of the bone matrix, the ruffled border secretes enzymes and protons after attachment to the bone. Cathepsin K activity is required to start actin ring formation and, thus, activation of OCL [54]. H^+^-ATPase is a potential mediator for the transfer of vesicules from microtubules to cortical actin network, via its direct binding to actin [55]. Mutations in the gene that encode for the a3 subunit of H^+^-ATPase cause osteopetrosis, due not only to a defect in acidification but also in ruffled border formation [56]. It has been shown that disruption in the actin ring by nitrogen-containing alendronate results in a reduced localization of MMP-9 in this structure, secretion of active form of MMP-9 and less OCL migration [57]. In agreement with the inhibition of osteoclastogenesis induced by IP6 treatment, a decrease on the resorption activity on dentin discs of both, human primary osteoclasts and OCL induced from RAW 264.7 cells was observed.

In order to study OCL resorption independently of OCL formation, cells were treated with IP6 once mature OCL had formed. OCL derived from RAW 264.7 cells treated with IP6 not only showed an increase in the mRNA levels of the four major players needed for the resorbing activity of OCL but they also developed higher bone resorption activity, with a significant increase in the resorbed area. However, when IP6 treatment was given to mature human osteoclasts, in contrast to the results obtained with the RAW264.7 cell line, a significant decrease in the resorbed area on dentin discs was observed. This difference could be explained by the heterogeneity of human primary osteoclasts, on one hand we cannot rule out that all the cells were at the same stage of maturation when IP6 treatment was given. On the other hand, this difference could be due to the use of non-purified PBMNC, without further isolation of CD14-positive monocytes. Nonetheless, this represents a more physiological system than the RAW 264.7 cell. As regards to the increased activity of mature OCL derived from RAW 264.7 cells after IP6 treatment, one possible explanation is that IP6 could increase the bone resorptive activity of mature OCL by inducing the activation of OCL survival-related signaling molecules such as c-Src/PI3K/Akt, Ras/ERK, and JNK/c-Jun signaling pathways. In this context, RANKL has been reported to act as an activating and survival factor for mature OCL, inducing Ras/ERK and JNK signaling pathways [58]. Further studies are currently ongoing to understand the inhibiton/activation of OCL survival pathways by IP6 and its target identification will be discussed in a near future.

BP have been attributed to directly inhibit bone resorption and to promote apoptosis of mature osteoclasts [47], [59], [60]. Instead, our data show that IP6 decreases osteoclastogenesis, increases the activation of mature OCL (in RAW 246.7 cells) and does not induce apoptosis in mature and precursor OCL (data not shown). Other molecules have recently been shown to inhibit osteoclastogenesis without inducing apoptosis, a novel mechanism that involves changes in posttranslational processing and trafficking of several proteins with known roles in osteoclast function has been proposed [61].

In the case of IP6, it is important to mention that IP6 is the precursor of inositol pyrophosphates, like IP7 and IP8, molecules that contain one or two high-energy pyrophosphate bonds [62], [63]. Up to 50% of IP6 has been estimated to be converted every hour to its pyrophosphorylated derivatives [62], pointing to the hypothesis that IP7 and IP8 may be responsible for many of the signaling-related functions previously attributed to IP6 [64]. Pyrophosphates regulate numerous processes including chemotaxis, telomere length, endocytic trafficking, exocytosis, and apoptosis (reviewed by Chakraborty *et al* [65]). Since fusion is required for osteoclastogenesis to take place [66] and endocytic trafficking of fusion-related proteins (i.e. DC-STAMP [67] is required for cell fusion, we hypothesize that IP6 could exert its effect by regulating cell fusion through the synthesis and action of inositol pyrophosphates IP7 and/or IP8. Efforts are currently underway in our laboratory to test that hypothesis in osteoclasts. Interestingly, we have shown a biphasic effect of IP6 on osteoclastogenesis, with a lower inhibition of osteoclastogenesis with concentrations higher than 1 µM, this finding could be explained by the efficiency of the conversion rate of IP6 to IP7/IP8. Thus, for high doses of IP6 the system might reach saturation and IP6 would not be further converted to IP7/IP8.

Although there are still numerous questions to be answered, in summary, our results demonstrate that IP6 inhibits osteoclastogenesis of human primary osteoclasts and of non-committed RAW 264.7 precursor cells. In addition, IP6 inhibits mature human primary osteoclasts function, although we did not find such effect on committed RAW 264.7 cells. Therefore, the present investigation demonstrates that IP6 may represent a novel type of selective inhibitor of osteoclasts and prove useful for the treatment of osteoporosis, which agrees with the inhibition of bone resorption by IP6 found in previous in vivo [28] and clinical reports [29], [30].
